# Systematic review and meta-analysis on the efficacy and safety of acupuncture for perimenopausal insomnia

**DOI:** 10.3389/fneur.2025.1649856

**Published:** 2025-08-11

**Authors:** ShiWei Song, Hao Chen, HongFang Fu

**Affiliations:** ^1^Department of Traditional Chinese Medicine, Sichuan Taikang Hospital, Chengdu, China; ^2^Chengdu University of Traditional Chinese Medicine, Chengdu, China; ^3^Department of Traditional Chinese Medicine, Sichuan Cancer Hospital and Institute, Chengdu, China

**Keywords:** acupuncture, perimenopausal insomnia, meta-analysis, systematic review, randomized controlled trials

## Abstract

**Background:**

Perimenopausal insomnia is a chronic physical and mental health disorder that plagues women. However, there are no systematic reviews or meta-analyses on the treatment of perimenopausal insomnia with acupuncture alone. Therefore, we conducted a meta-analysis to assess the efficacy and safety of acupuncture alone in improving perimenopausal insomnia.

**Methods:**

This study searched for randomized controlled trials on acupuncture treatment for perimenopausal insomnia from seven major literature databases in both Chinese and English: Web of Science, PubMed, the Cochrane Library, Embase, China National Knowledge Infrastructure, VIP database, and Wan-fang database. The quality of the studies was assessed according to the Cochrane Handbook for Systematic Reviews of Interventions. Meta-analysis was conducted using Rev Man 5.4 software.

**Results:**

The study comprised nine randomized controlled trials involving 968 people. The results showed that acupuncture was superior to the control group in improving the effective rate (OR: 3.30; 95% CI: 2.18–4.98; *p* < 0.00001), PSQI score (MD: −3.26; 95% CI: −4.62– −1.90; *p* < 0.00001), FSH (MD: −11.01; 95% CI: −15.39– −6.63; *p* < 0.00001), KMI score (*p* < 0.05), 5-HT (*p* < 0.05), NE (*p* < 0.05), MENQOL score (*p* < 0.05), early-wake score (*p* < 0.05), sleep actigraphy monitoring (*p* < 0.05), and Traditional Chinese Medicine symptom scores (*p* < 0.05) in patients with menopausal insomnia. The two groups had no significant differences in regulating serum E2 (MD: 7.70; 95% CI: 2.20–13.19; *p* = 0.06) and LH levels (MD: −5.42; 95% CI: −9.46– −1.37; *p* = 0.009).

**Conclusion:**

Acupuncture treatment is significantly effective for patients with perimenopausal insomnia. However, large-sample, multi-center, long-term follow-up trials should be conducted to obtain more reliable results. Considering the particularities of acupuncture treatment, actively constructing a real-world acupuncture clinical research paradigm will bring more authentic, rich, and practical research outcomes to clinical practitioners.

**Systematic review registration:**

https://www.crd.york.ac.uk/PROSPERO/view/CRD42024621267, identifier CRD42024621267.

## 1 Introduction

Perimenopausal insomnia is a common chronic disease in clinical practice that has plagued female patients for a long time. Along with an aging society, long-term insomnia severely affects the physical and mental health of women and increases the social burden (1, 2). Relevant reports indicate that the prevalence rate among premenopausal women is between 16 and 42% (3), affecting their quality of life. With the onset of perimenopause, the risk of sleep disorders significantly increases (4, 5). Moreover, the probability of insomnia in the late perimenopausal period is about 1.3 times that of the early stage (6). There is also a noticeable difference in sleep disorders among perimenopausal women in different countries, with Caucasians at 40%, the prevalence rate in the United States ranging from 30 to 40% (4), and the prevalence rate in China significantly higher than in the United States, exceeding 50% (7). In contrast, other Asian countries such as Japan are close to 30%, and South Korea is around 15% (8).

Perimenopausal insomnia features light sleep, insufficient sleep duration, difficulty falling asleep, frequent dreams, and difficulty returning to sleep after waking up, accompanied by symptoms such as dizziness, chest tightness, palpitations, anxiety, depression, and irritability (9–11). The condition is characterized by its propensity for recurring episodes, its chronic nature, and the considerable difficulty of its cure. The current main medical treatment method is hormone replacement therapy combined with sedative/hypnotic drugs (12). Although this method can temporarily alleviate sleep disorders, long-term use has significant dependency and can lead to severe side effects (13). Consequently, there is an increasing inclination among the population toward the utilization of complementary and alternative therapies, with acupuncture being a particularly popular choice (14).

Acupuncture, as an affordable, convenient, effective, and minimal side-effect external treatment in traditional Chinese medicine, has been widely used in the treatment of perimenopausal insomnia and has demonstrated clear therapeutic effects in a series of study (15–17). However, in the past decade, researchers have only conducted a few meta-analyses on acupuncture for perimenopausal insomnia, and no one has conducted a meta-analysis comparing pure acupuncture with pure Western medicine. Although sedative-hypnotics are recommended as first-line therapy for perimenopausal insomnia, roughly half of the patients discontinue them because of contraindications, adverse effects, or high relapse rates. Among non-pharmacological options, acupuncture has shown promise; however, existing systematic reviews often conflate acupuncture with “usual care” or “combination treatments” without isolating comparisons against purely Western pharmacotherapy. This conflation obscures the true effect attributable to acupuncture alone. Therefore, clarifying the net effect of acupuncture relative to conventional Western medications is an urgent priority for clinical decision-making. Therefore, this study synthesizes the latest research findings and, for the first time, evaluates the efficacy difference between pure acupuncture and pure Western medicine in treating perimenopausal insomnia through a comprehensive meta-analysis. The present study provides empirical evidence to inform a more comprehensive and objective understanding of the role of acupuncture in perimenopausal insomnia.

## 2 Materials and methods

### 2.1 Study registration

This study employed the latest PRISMA 2020 criteria, and we explicitly declare that the review was conducted in accordance with the PRISMA 2020 guidelines. A complete scheme for this study was registered in the Prospective Register of Systematic Reviews (PROSPERO): No. CRD42024621267.

### 2.2 Inclusion criteria

Patient: Patients meet the international diagnostic criteria for perimenopausal insomnia, regardless of race, color of skin, and source of the patients. Intervention: The experimental group only received acupuncture treatment. Comparison: The control group received Western treatment. Outcome: The effective rate, Pittsburgh Sleep Quality Index score, Estradiol, Follicle-stimulating hormone, Luteinizing hormone, Kupperman menopause index score, 5-hydroxy tryptophan, norepinephrine, Menopause-specific Quality of Life Questionnaire score, early-wake score, sleep actigraphy monitoring, Traditional Chinese Medicine symptom scores. Study design: The study included only randomized controlled trials published in English or Chinese on acupuncture treatment for perimenopausal insomnia.

### 2.3 Exclusion criteria

(1) Research on animal experiments; (2) Case reports, reviews, letters, and comments; (3) Combined with other diseases; (4) Treatment group with acupuncture and other therapies; (5) Full text not available; (6) Master’s and doctoral dissertations; (7) Randomized control is unclear; (8) Incomplete literature data; (9) Systematic review and pharmacological study; (10) Repeatedly published literature.

### 2.4 Search strategy

Computer retrieval of the following seven databases: Web of Science, PubMed, the Cochrane Library, Embase, China National Knowledge Infrastructure, VIP database, and Wan-fang database. All from the inception to December 5, 2024. In addition, the manual retrieval of relevant materials is conducted. While also collecting gray literature and tracing the references of included documents to supplement the acquisition of related literature. The search terms and keywords included “perimenopause,” “perimenopausal,” “menopause,” “menopausal,” “climacteric,” “climacterium,” “insomnia,” “sleepless,” “sleep disorder,” “sleep initiation dysfunction,” “sleep initiation and maintenance disorders,” “sleep maintain dysfunction,” “lose sleep,” “acupoint,” “electroacupuncture,” “pharmacopuncture,” “acupuncture,” “needling,” “acupuncture therapy,” “needling therapy,” “acupuncture treatment,” “manual acupuncture,” “auricular acupuncture,” “acupuncture point,” “Randomized controlled trial” and “clinical trials.” Using PubMed as an example, the search strategy is shown in Table 1 below.

**Table 1:** PubMed: session results.

Number	Search details	Results
#1	“Acupuncture” [MeSH Terms]	2,103
#2	“acupuncture” [Title/Abstract] OR “electroacupuncture” [Title/Abstract] OR “pharmacopuncture” [Title/Abstract] OR “acupuncture treatment” [Title/Abstract] OR “acupuncture therapy” [Title/Abstract] OR “needling therapy” [Title/Abstract] OR “needling” [Title/Abstract] OR “manual acupuncture” [Title/Abstract] OR “auricular acupuncture” [Title/Abstract] OR “acupuncture point” [Title/Abstract] OR “acupoint” [Title/Abstract]	37,890
#3	#1 OR #2	38,098
#4	“Sleep Initiation and Maintenance Disorders” [MeSH Terms]	19,585
#5	(“sleep initiation” [Title/Abstract] AND “maintenance disorders” [Title/Abstract]) OR “insomnia” [Title/Abstract] OR “sleepless” [Title/Abstract] OR “sleep disorder” [Title/Abstract] OR “sleep initiation dysfunction” [Title/Abstract] OR (((“Sleep” [MeSH Terms] OR “Sleep” [All Fields] OR “sleeping” [All Fields] OR “sleeps” [All Fields] OR “sleep s” [All Fields]) AND (“maintainance” [All Fields] OR “maintained” [All Fields] OR “maintaining” [All Fields] OR “maintains” [All Fields] OR “Maintenance” [MeSH Terms] OR “Maintenance” [All Fields] OR “maintain” [All Fields])) AND “dysfunction” [Title/Abstract]) OR (“lose” [All Fields] AND “Sleep” [Title/Abstract])	39,345
#6	#4 OR #5	44,468
#7	“Perimenopause” [MeSH Terms]	1770
#8	“perimenopausal” [Title/Abstract] OR “menopause” [Title/Abstract] OR “menopausal” [Title/Abstract] OR “climacteric” [Title/Abstract] OR “climacterium” [Title/Abstract] OR “perimenopause” [Title/Abstract]	67,458
#9	#7 OR #8	67,588
#10	#3 AND #6 AND #9	47
#11	((“Acupuncture” [MeSH Terms] OR (“Acupuncture” [Title/Abstract] OR “electroacupuncture” [Title/Abstract] OR “pharmacopuncture” [Title/Abstract] OR “acupuncture treatment” [Title/Abstract] OR “acupuncture therapy” [Title/Abstract] OR “needling therapy” [Title/Abstract] OR “needling” [Title/Abstract] OR “manual acupuncture” [Title/Abstract] OR “auricular acupuncture” [Title/Abstract] OR “acupuncture point” [Title/Abstract] OR “acupoint” [Title/Abstract])) AND (“Sleep Initiation and Maintenance Disorders” [MeSH Terms] OR ((“sleep initiation” [Title/Abstract] AND “maintenance disorders” [Title/Abstract]) OR “insomnia” [Title/Abstract] OR “sleepless” [Title/Abstract] OR “sleep disorder” [Title/Abstract] OR “sleep initiation dysfunction” [Title/Abstract] OR (((“Sleep” [MeSH Terms] OR “Sleep” [All Fields] OR “sleeping” [All Fields] OR “sleeps” [All Fields] OR “sleep s” [All Fields]) AND (“maintainance” [All Fields] OR “maintained” [All Fields] OR “maintaining” [All Fields] OR “maintains” [All Fields] OR “Maintenance” [MeSH Terms] OR “Maintenance” [All Fields] OR “maintain” [All Fields])) AND “dysfunction” [Title/Abstract]) OR (“lose” [All Fields] AND “Sleep” [Title/Abstract]))) AND (“Perimenopause” [MeSH Terms] OR (“perimenopausal” [Title/Abstract] OR “menopause” [Title/Abstract] OR “menopausal” [Title/Abstract] OR “climacteric” [Title/Abstract] OR “climacterium” [Title/Abstract] OR “Perimenopause” [Title/Abstract]))) AND (randomizedcontrolledtrial [Filter])	18

### 2.5 Outcome assessment indicators

Primary outcome measures: The effective rate and the Pittsburgh Sleep Quality Index score.

Secondary outcome assessment indicators: serum Estradiol, the Follicle-stimulating hormone, the Luteinizing hormone, Kupperman menopause index score, 5-hydroxy tryptophan, Norepinephrine, Menopause-specific quality of life questionnaire score, early-wake score, sleep actigraphy monitoring, and Traditional Chinese Medicine symptom scores.

### 2.6 Literature screening and data extraction

EndNoteX9 was used to enter all collected articles. After excluding duplicate literature, two investigators preliminarily screened all retrieved literature based on the inclusion and exclusion criteria, excluding ineligible documents. For the remaining literature, they read the full text again and conducted a detailed analysis to assess the study population, study type, primary and secondary outcome measures, etc. Then, we excluded documents that did not meet the inclusion criteria. Finally, two investigators independently extracted basic data from each eligible article and input it into a standardized Excel spreadsheet. The entire process was conducted by two members, and any disagreements were resolved through consultation with a third researcher to facilitate consensus.

### 2.7 Risk bias and quality assessment

The methodological quality of the included trials was assessed with the help of the Cochrane Collaboration’s risk of bias assessment tool. There are seven items in total: (1) Randomization method, detailing the method used to generate the random allocation sequence to assess comparability between groups, judged low risk only when the report explicitly stated “random” and described the sequence-generation method; (2) Allocation concealment, determining whether the assignment of interventions was predictable, judged low risk only when the use of sealed opaque envelopes, central telephone, or an online system was described; (3) Description of the method used to implement blinding for subjects or trial personnel, owing to the nature of acupuncture, blinding of acupuncturists is impossible; trials using sham acupuncture with successful participant blinding were rated “some concerns”; (4) Blinding for outcome measurement, low risk only when the manuscript stated that assessors were unaware of group allocation; (5) Integrity of the data; (6) Selective reporting of study results; (7) Other sources of bias that should be addressed in the full text.

### 2.8 Statistical analysis

The software used is the Rev Man 5.4 version provided by the Cochrane Collaboration. For binary outcomes and continuous outcomes, the odds ratio and mean difference are used as measures of effectiveness, with 95% confidence intervals. If there is no statistically significant heterogeneity between studies, the analysis is undertaken using a fixed-effect model; if there is a statistically significant heterogeneity, a random-effect model is employed. The degree of heterogeneity among the studies is measured using the *I*^2^ statistic. When the *I*^2^ > 50%, it indicates significant heterogeneity, and the higher the value, the greater the degree of heterogeneity. When necessary, analyze the sources of heterogeneity and perform a sensitivity analysis. *p* < 0.05 indicates that the difference is statistically significant. If any pooled outcome is still reported by≤2 studies, quantitative synthesis will be abandoned in favor of a narrative summary.

## 3 Results

### 3.1 Literature search results

**Figure 1:**
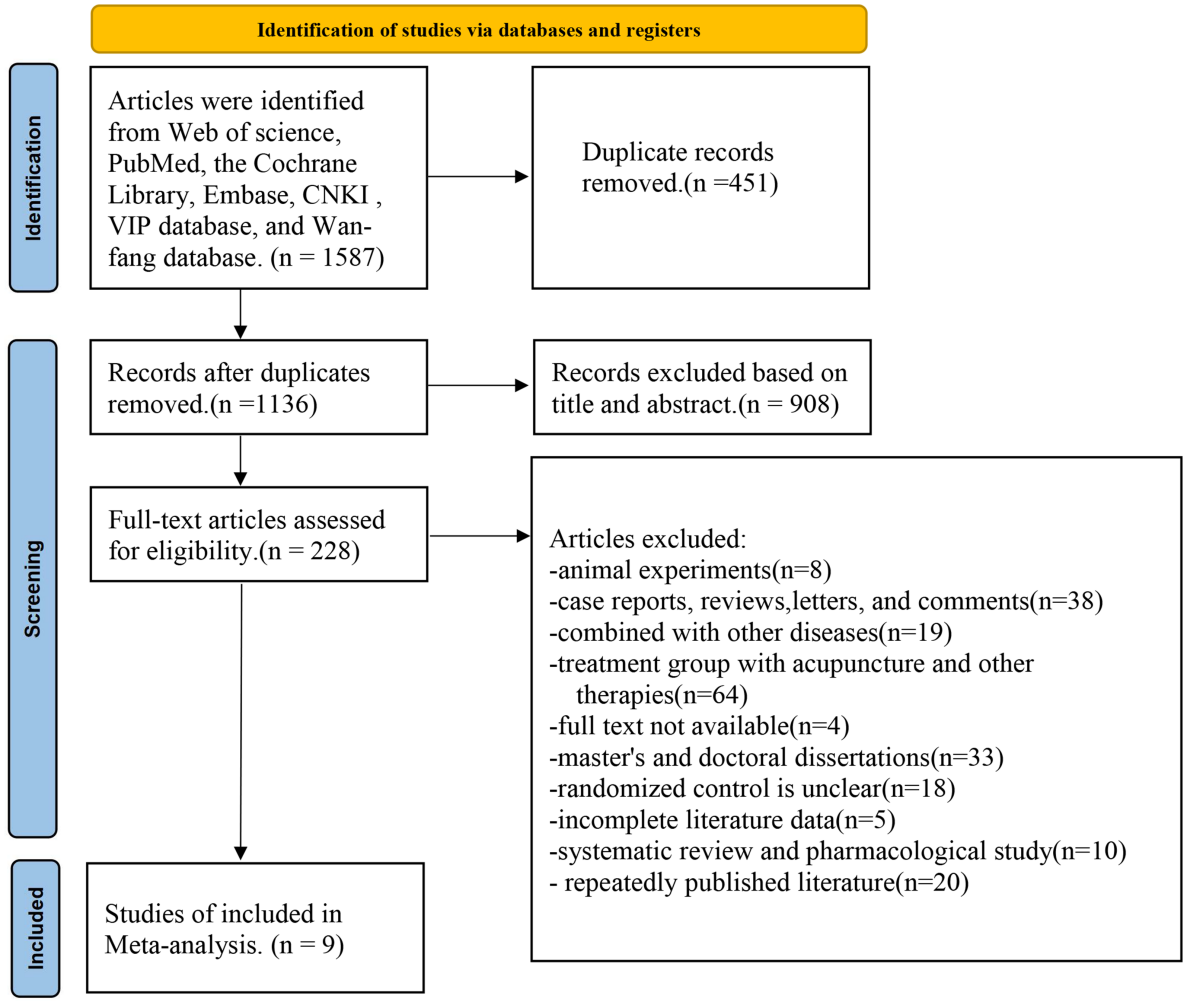
Literature screening process.

By searching seven public databases using predefined thematic terms, 1,587 articles were identified. After excluding 451 duplicate publications, A total of 908 articles deemed to be irrelevant were excluded based on their titles and abstracts, leaving 228 articles for further assessment. The 219 excluded articles included: research on animal experiments (*n* = 8), case reports, reviews, letters, and comments (*n* = 38), combined with other diseases (*n* = 19), treatment group with acupuncture and other therapies (*n* = 64), full text not available (*n* = 4), master’s and doctoral dissertations (*n* = 33), randomized control is unclear (*n* = 18), incomplete literature data (*n* = 5), systematic review and pharmacological study (*n* = 10), repeatedly published literature (*n* = 20). This study ultimately included a total of 9 articles, involving 968 female patients, to assess the efficacy and safety of pure acupuncture compared to pure Western medicine in the treatment of perimenopausal insomnia (Figure 1).

The general information and characteristics of 9 studies are presented in Table 2 (18–26). The present study comprised a total of nine articles. The publication period is between 2014 and 2023. Nine articles involved 968 female patients, with 483 cases in the experimental group and 485 cases in the control group. The minimum sample size was 53 cases, and the maximum sample size was 240 cases. The experimental group primarily utilized acupuncture alone, while the control group primarily relied on oral sedatives. The fundamental characteristics of the articles under consideration are set out in Table 2.

**Table 2:** Characteristics of 9 studies.

Study	Study design	Sample size (T:C)	Age (year), Mean ± SD	Disease duration(year), Mean ± SD	Intervention	Comparison	Treatment duration (days)	Follow-up (months)	Outcomes
Li et al. (20)	RCT	T:120 C:120	T:50.2士2.20 C:49.8士2.3	T:1.1士0.2 C:0.8士0.2	Acupuncture	Estazolam tablets	30	NM	The effective rate, PSQI score
Wang et al. (22)	RCT	T:28 C:28	T:50.28士2.32 C:50.37士2.31	T:2.7士1.2 C:2.6士1.3	Acupuncture	Estazolam tablets	28	NM	PSQI score
Li and Wang (19)	RCT	T:60 C:62	T:51士4 C:50士4	T:0.93士0.43 C:0.85士0.44	Acupuncture	Alprazolam tablets	63	NM	The effective rate, PSQI score, E2, FSH, LH
Song et al. (21)	RCT	T:106 C:106	T:46.72士2.13 C:50.73士3.65	T:2.73士2.14 C:3.24士1.58	Acupuncture	Lorazepam tablets	30	NM	The effective rate, E2, FSH
Yang et al. (24)	RCT	T:30 C:30	T:50.08士2.17 C:50.12士2.26	T:1.62士0.51 C:1.54士0.48	Acupuncture	Alprazolam tablets	28	NM	The effective rate, PSQI score
Guo et al. (18)	RCT	T:30 C:30	T:49.83士3.65 C:50.20士4.10	T:3.04士1.13 C:2.83士1.23	Acupuncture	Estazolam tablets	56	NM	The effective rate, PSQI score, KMI score, E2, 5-HT, NE
Yan et al. (23)	RCT	T:42 C:43	T:51.5士4.6 C:50.08士2.17	T:0.74士0.47 C:0.76士0.50	Acupuncture	Estazolam tablets	84	NM	The effective rate, PSQI score, MENQOL score
Zhu et al. (26)	RCT	T:27 C:26	T:50士3 C:50士2	T:0.77士0.4 C:0.77士0.38	Acupuncture	Oryzanol Tablets	28	NM	The effective rate, PSQI score, early-wake score, sleep actigraphy monitoring score
Zhao et al. (25)	RCT	T:40 C:40	T:49.85士2.46 C:50.17士2.71	T:0.43士0.17 C:0.50士0.19	Acupuncture	Estazolam tablets	28	NM	The effective rate, PSQI score, TCM symptom score, E2, FSH, LH

T, intervention group; C, comparison group; NM, not mention; RCT, randomized controlled trial; PSQI, Pittsburgh Sleep Quality Index; E2, Estradiol; FSH, follicle-stimulating hormone; LH, luteinizing hormone; KMI, Kupperman menopause index; 5-HT, 5-hydroxytryptopha; NE, norepinephrine; MENQOL, Menopause-specific Quality of Life Questionnaire; TCM, Traditional Chinese Medicine.

Out of these, 8 studies assessed the effectiveness of the treatment, 8 analyzed the Pittsburgh Sleep Quality Index score, 4 measured serum estradiol, 3 analyzed the Follicle-Stimulating Hormone, and 2 reported on the Luteinizing Hormone. Additionally, other secondary outcome measures, including Kupperman menopause index score, 5-hydroxy tryptophan, norepinephrine, Menopause-Specific Quality of Life score, early-wake score, sleep actigraphy monitoring, and Traditional Chinese Medicine symptom scores, were each reported in only one piece of literature.

### 3.2 Methodological and reporting quality

**Figure 2:**
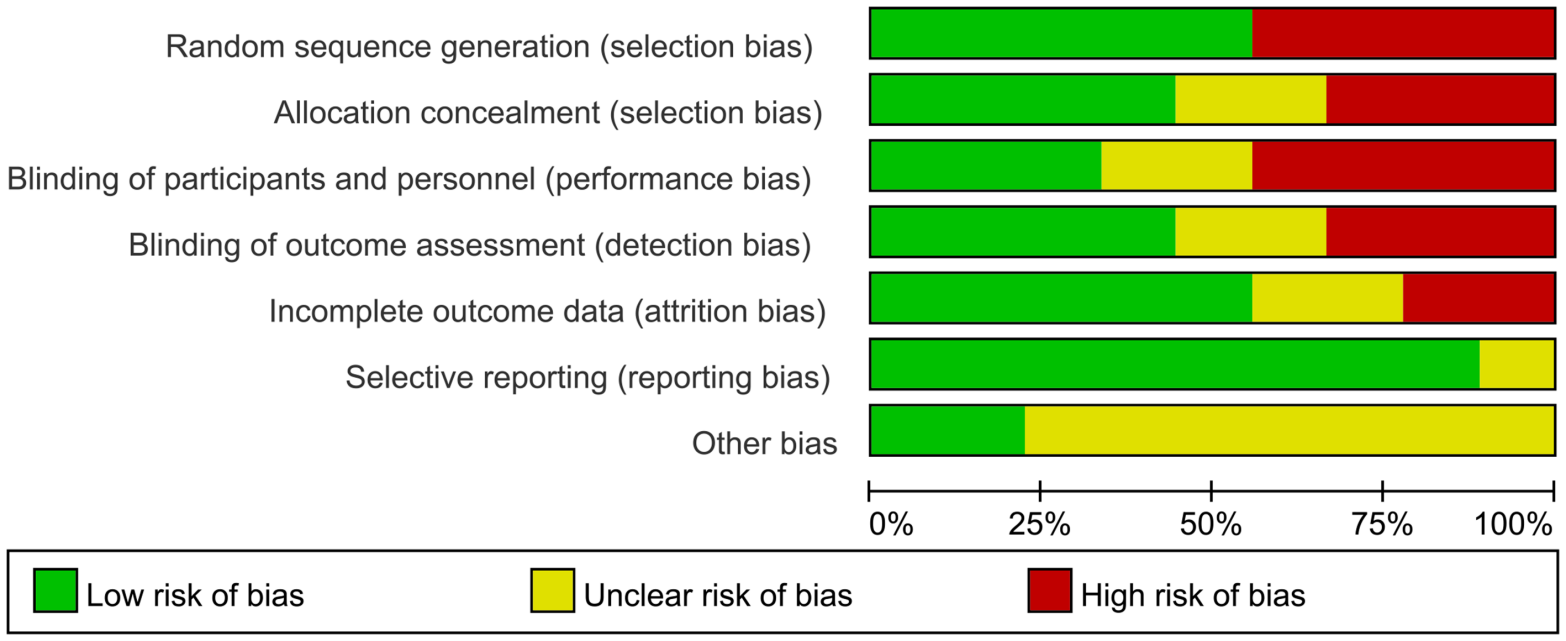
Assessment of the risk of bias of 9 articles.

**Figure 3:**
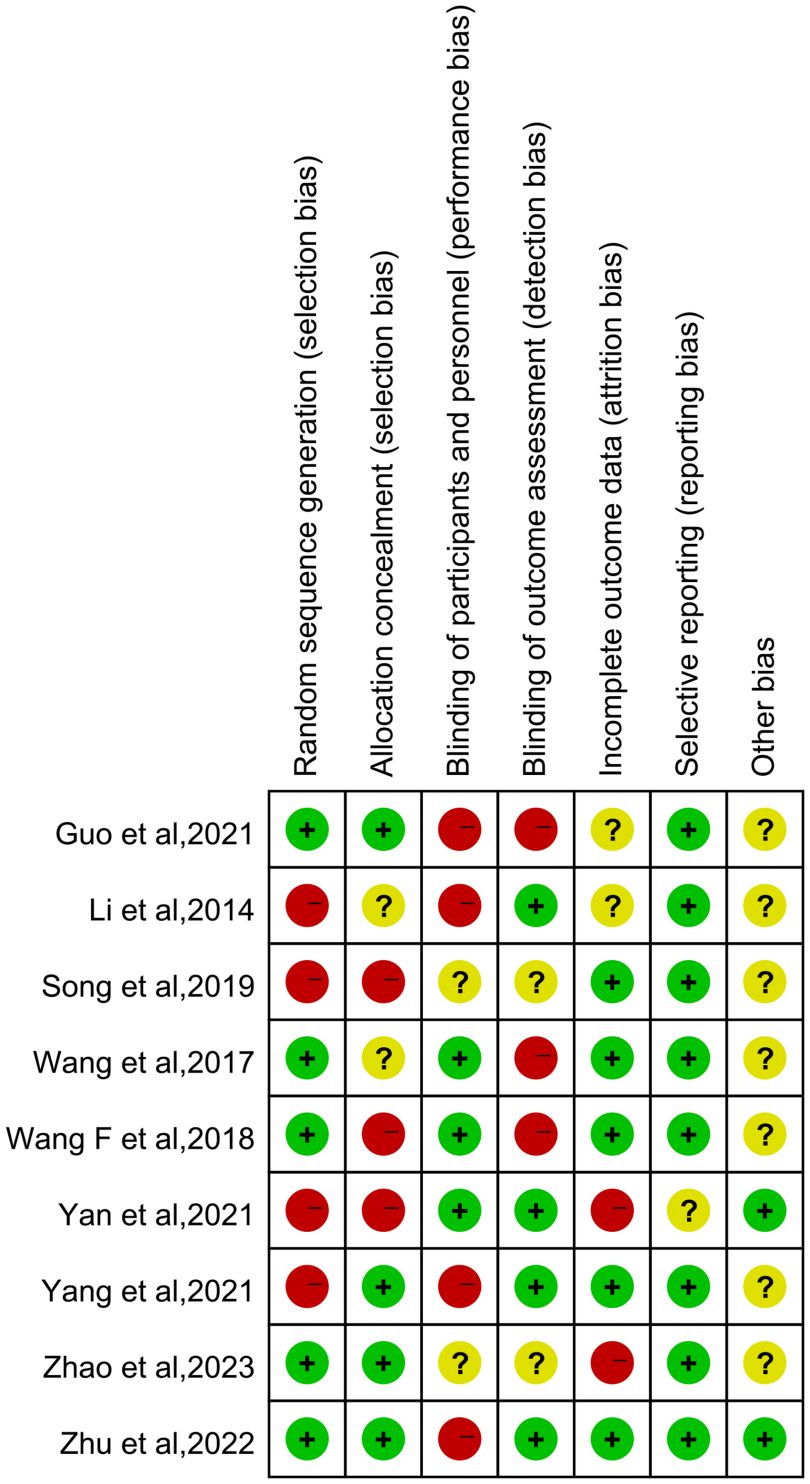
The overall risk bias assessment chart of nine literature.

The results show that 5 studies (18, 19, 22, 25, 26) used a random number table, presenting a low risk in the generation of random sequences. Regarding the concealment of allocation schemes, nearly half of the studies (18, 24–26) described whether the study implemented allocation concealment or specific schemes. In terms of blinding for subjects and researchers, 3 studies (19, 22, 23) described how researchers or subjects were unblinded. Concerning the evaluation of outcome assessment blinding, a total of four articles were categorized as low risk (20, 23, 24, 26). In terms of the integrity of outcome data, more than half of the studies (19, 21, 22, 24, 26) fully reported outcome measures. In the assessment of the risk of bias in reporting, it was determined that only one study was deemed to have an indeterminate risk (23). In the absence of further research, the prevailing literature was largely equivocal concerning the existence of other biases (18–22, 24, 25). Figures 2, 3, respectively, display the risk of bias graph for individual studies and a summary of the risk of bias graph.

### 3.3 The effective rate

**Figure 4:**
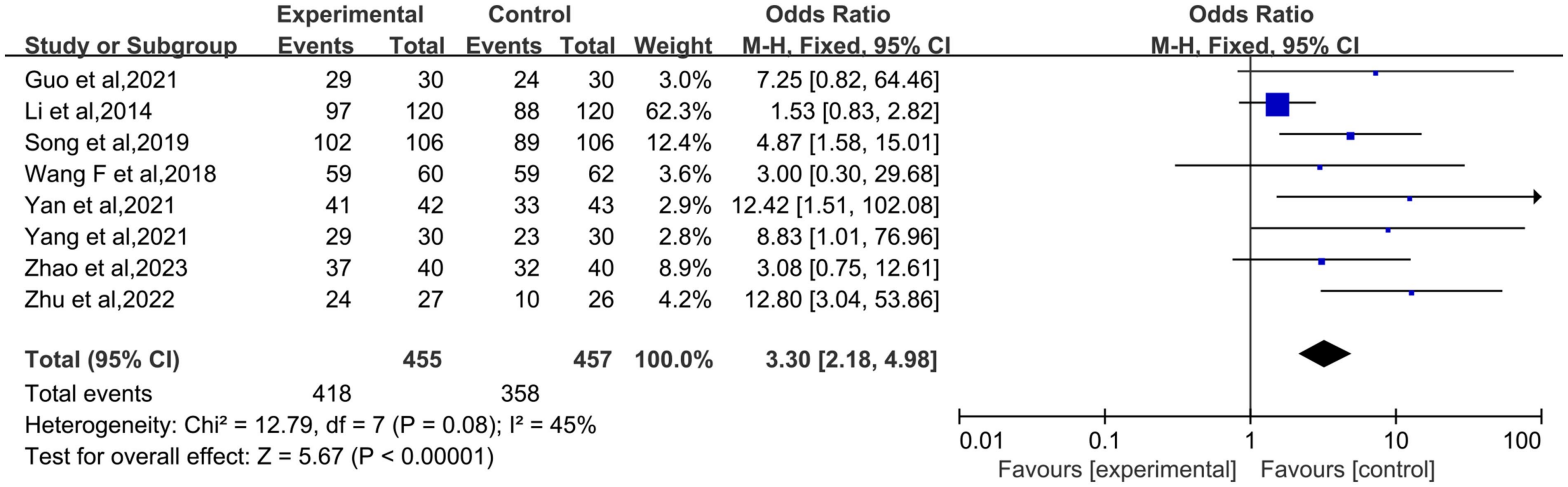
Forest plot of acupuncture efficiency for perimenopausal insomnia.

Eight studies (18–21, 23–26) reported the effective rate of acupuncture treatment for perimenopausal insomnia. Through the heterogeneity analysis of the effective rate, following an analysis of the extant literature, it was determined that there was a paucity of heterogeneity. Consequently, a fixed-effect model was selected (*I*^2^ = 45%; *p* = 0.08). In terms of the effective rate, the efficacy of pure acupuncture treatment for perimenopausal insomnia was superior to the control group (OR: 3.30; 95% CI: 2.18–4.98; *p* < 0.00001). The specific results can be observed in Figure 4.

### 3.4 Pittsburgh sleep quality index score

**Figure 5:**
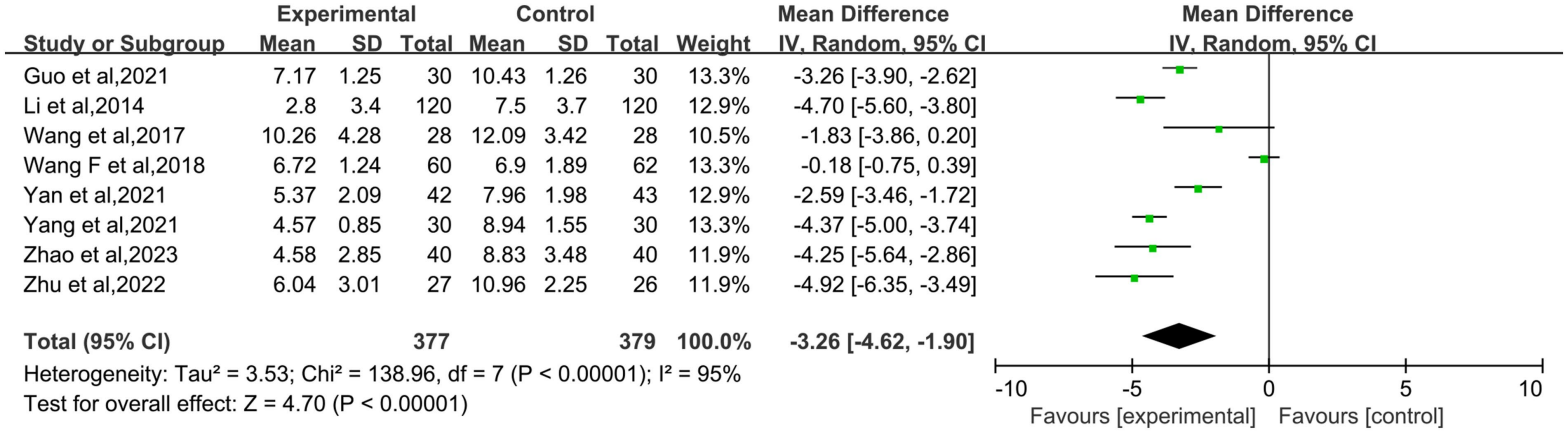
Forest plot of meta-analysis on PSQI score for perimenopausal insomnia.

A total of eight studies (18–20, 22–26) were included in the analysis, with the Pittsburgh Sleep Quality Index score being the primary metric of interest. The literature showed statistical heterogeneity (*I*^2^ = 95%; *p* < 0.00001), and a random-effects model was employed to ascertain the combined effect (MD: −3.26; 95% CI: −4.62– −1.90; *p* < 0.00001, Figure 5). The results demonstrated that acupuncture treatment for perimenopausal insomnia significantly reduced the PSQI scores compared to the control group, with the difference being statistically significant.

### 3.5 Serum estradiol

**Figure 6:**
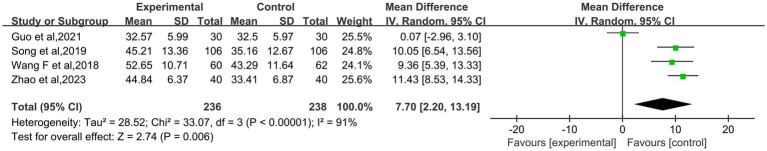
Forest plot of meta-analysis on serum Estradiol for perimenopausal insomnia.

Four studies (18, 19, 21, 25) reported changes in serum Estradiol levels in 474 patients. The analysis was conducted utilizing a random-effects model (MD: 7.70; 95% CI: 2.20–13.19; *p* = 0.06, Figure 6). There was no homogeneity among the studies (*p* < 0.00001, *I*^2^ = 91%). The results showed that there was no statistically significant difference in increasing E2 levels between acupuncture treatment for perimenopausal insomnia and the control group.

### 3.6 Follicle-stimulating hormone

**Figure 7:**
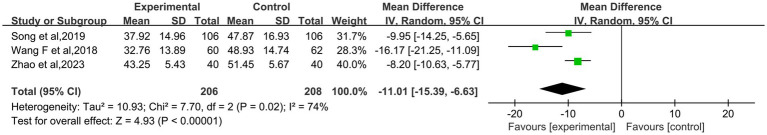
Forest plot for FSH in two groups.

Three studies (19, 21, 25) documented the changes in Follicle-stimulating hormone levels, and the literature exhibited statistical heterogeneity (*p* = 0.02, *I*^2^ = 74%). The pooled effect was analyzed using a random-effects model (MD: −11.01; 95% CI: 15.39– −6.63; *p* < 0.00001, Figure 7), and the results demonstrated that acupuncture treatment in the experimental group significantly reduced FSH levels.

### 3.7 Luteinizing hormone

**Figure 8:**
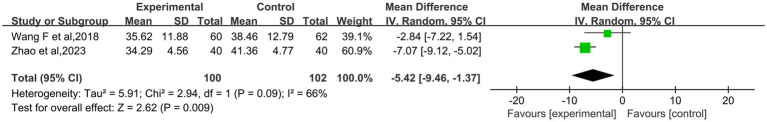
Forest plot of LH levels.

Only two articles (19, 25) reported on Luteinizing hormone levels, a heterogeneity test was conducted, yielding a *p*-value of 0.09 and an I^2^ value of 66%. This finding indicates a statistically significant difference between the two studies, thus necessitating the implementation of a random-effects model (MD: −5.42; 95% CI: −9.46– −1.37; *p* = 0.009, Figure 8). The results show that the change in LH levels in the experimental group of perimenopausal insomnia patients compared to the control group was not statistically significant, and it is not possible to conclude that acupuncture is more effective than Western medicine in improving LH levels.

### 3.8 Other secondary outcome assessment indicators

One article (18) reported on the Kupperman menopause index score, 5-hydroxy tryptophan, and norepinephrine, and found that there was a statistically significant difference between the acupuncture group and the control group (*p* < 0.05). Moreover, a further article (23) conducted a test on the Menopause-Specific Quality of Life Questionnaire score and found that both groups showed improvement in the two indicators after treatment, with acupuncture treatment being superior to Western medicine treatment (*p* < 0.05). One article (26) reported that the treatment group showed better improvement in early-wake score and sleep actigraphy monitoring compared to the control group (*p* < 0.05). Regarding Traditional Chinese Medicine (TCM) symptom scores, the results indicated that only one report (25) indicated a more significant reduction in the observed group compared to the control group (*p* < 0.05).

### 3.9 Security evaluation

In eight studies, no adverse reactions were observed when the experimental and control groups were compared (18–22, 24–26). Only one study found adverse reactions: Yan (23) reported that in the treatment group, there were 3 cases of needle fainting and 1 case of subcutaneous hematoma; in the control group, there was 1 case of dizziness and fatigue, and 2 cases of somnolence. All adverse reactions resolved spontaneously without special treatment and did not affect the entire treatment process. As only one study reported adverse events, current evidence is insufficient to claim that acupuncture is safer than Western medication; this needs to be confirmed in future research.

### 3.10 Sensitivity analysis

A sensitivity analysis was conducted on the primary outcome measure, the PSQI score, using a method of excluding studies on an individual basis. After excluding each study, there was no significant change in heterogeneity, suggesting that the sources of heterogeneity may be due to differences in acupuncture point selection, techniques, needle depth, and interventions in the control group.

### 3.11 Publication bias

This study only included nine articles, which does not meet the criteria for creating a funnel plot. Consequently, a funnel plot bias test was not conducted, and there is a possibility of bias in the efficiency of the study results.

## 4 Discussion

### 4.1 Summary of major results

This analysis included a total of 9 articles involving 968 women with menopausal insomnia. The results showed that acupuncture was superior to the control group in improving the effective rate, PSQI score, FSH, KMI score, 5-HT, NE, MENQOL score, early-wake score, sleep actigraphy monitoring, and Traditional Chinese Medicine symptom scores in patients with menopausal insomnia. No significant differences were observed between the two groups in regulating serum E2 and LH levels. The results suggested that acupuncture can achieve a beneficial effect on the treatment of menopausal insomnia. In conclusion, the present study has demonstrated that acupuncture is an efficacious treatment for insomnia in perimenopausal patients. This conclusion is restricted to the limited outcomes evaluated by the included studies; the effects on other clinically important endpoints require validation through additional high-quality research.

Perimenopause is one of the significant stages in a woman’s life, and research indicates that the prevalence of insomnia among Chinese perimenopausal women ranges from 47 to 65% (27–29). As the global population ages rapidly, insomnia has become one of the primary health concerns for all perimenopausal women. Insomnia during the perimenopausal period has turned into a significant public health issue, exerting a substantial impact on daily living activities (30). The etiology of perimenopausal insomnia is quite complex. Modern clinical studies demonstrated that perimenopausal insomnia is associated with factors such as aging, mental stress, neuroendocrine changes, and hormonal levels. A multitude of factors may contribute to the development of the condition, including but not limited to psychological factors, lifestyle habits, the social environment, and air pollution (31). Currently, there is no consensus on the exact pathogenesis of this condition. Western medical theory posits that the pathogenesis of perimenopausal insomnia is primarily due to the decline in ovarian function, accompanied by a decrease in sex hormone levels. The decline in ovarian function leads to an imbalance in the hypothalamic–pituitary-ovarian axis, resulting in a range of symptoms. Insomnia has been identified as a primary symptom of perimenopausal syndrome and is largely caused by hormonal disruptions in women’s bodies (32). The mainstream treatments for perimenopausal insomnia in Western medicine include pharmacological and non-pharmacological approaches. Pharmacological treatments primarily involve oral sedatives and hypnotics, as well as hormone replacement therapy. Non-pharmacological treatments encompass aromatherapy, hypnosis, psychotherapy, cognitive-behavioral therapy, music therapy, and so on (33). Western medical drug treatments for perimenopausal insomnia have limitations such as significant side effects and the inability to be taken in large doses for long periods. In contrast, integrative medicine and complementary therapies have distinct advantages. Acupuncture, as a characteristic treatment method of traditional Chinese external therapies, has the advantages of a wide range of indications, significant efficacy, cost-effectiveness, and few adverse reactions. Acupuncture is a well-established practice that has been employed extensively in the treatment of a wide range of diseases (34).

Traditional Chinese medicine believes that insomnia is closely related to the disharmony of Yin and Yang in the human body. Perimenopausal women often experience disharmony in the Chong and Ren meridians, deficiency of liver and kidney Yin, and insufficiency of essence and blood, leading to an imbalance of Yin and Yang. This results in the failure of Yang to enter Yin, causing insomnia. Acupuncture of related acupoints can communicate the meridian Qi and blood, regulate the Yin and Yang of the viscera, guide Yang into Yin, and bring the body to a state of balanced Yin and hidden Yang, thereby improving sleep symptoms (35). In recent years, clinical studies and research on the pathological mechanisms of acupuncture for treating perimenopausal insomnia have gradually increased (36–38). Contemporary research has demonstrated the capacity of acupuncture to regulate the hypothalamic–pituitary-ovarian axis and enhance hormone secretion levels in patients, thereby improving insomnia symptoms (36). Moreover, the extant literature suggests that acupuncture may work through a bidirectional regulatory mechanism to stimulate multiple functional systems in the body. By modulating the system’s corrective functions, it aims to improve insomnia (39). Animal experimental research has found that acupuncture can restore the sleep–wake cycle of PCPA-induced insomnia rats by upregulating the expression of hippocampal 5-HT1A total RNA and downregulating the expression of hippocampal 5-HT2A total RNA. It can thus be concluded that acupuncture plays a role in the treatment of insomnia (40). In recent years, research on acupoints related to perimenopausal insomnia has emerged continuously. Commonly used acupoints include San Yin Jiao (SP6), Shen Men (HT7), Xin Shu (BL15), Bai Hui (GV20), and Tai Xi (KI3) (41, 42). The selection of these acupoints is based on traditional Chinese medicine theory, aiming to regulate the body’s Qi and blood as well as the balance of Yin and Yang. The long-term efficacy of acupuncture treatment is more pronounced than that of Western medications, with significantly reduced side effects. This makes it worthy of strong promotion by researchers. Some researchers have reported in systematic reviews on acupuncture for insomnia that stimulation of specific acupoints can modulate protein expression, and via vagal regulation, influence neuroendocrine homeostasis to improve sleep. Additionally, acupuncture was found to increase levels of sleep-related neurotransmitters such as serotonin and *γ*-aminobutyric acid while decreasing the concentration of sleep-inhibitory neurotransmitters like norepinephrine in the brain. These findings provide direct evidence supporting the biological plausibility of acupuncture for managing perimenopausal insomnia (43). It must be explicitly stated that the cited Traditional Chinese Medicine theories and animal experiments serve only as theoretical speculation or preclinical evidence, and their clinical relevance still awaits confirmation through further human trials.

### 4.2 Strengths, limitations, and comparison with previous systematic reviews

Acupuncture’s efficacy in improving PMI compared to sham acupuncture has been confirmed in previous systematic evaluations. This is the first time a systematic review and meta-analysis has been conducted to assess the effectiveness and safety of pure acupuncture compared with pure Western medication in improving PMI. In past studies, the utilization of acupuncture has historically been largely confined to a complementary role, often employed in conjunction with conventional Western medical interventions, as a phase in a comprehensive treatment plan. Our study specifically addresses this issue, considering acupuncture as a mainstream therapeutic approach, and for the first time, evaluates the efficacy of acupuncture alone in improving perimenopausal insomnia.

In the past decade, systematic reviews and meta-analyses have been relatively scarce, and the treatment groups in studies often include other traditional Chinese external therapies, such as moxibustion, electroacupuncture, Tuina massage, Gua Sha therapy, ear acupressure, point application, and catgut embedding. Furthermore, practices such as Tai Chi, Baduanjin, and Qigong, which are traditional Chinese exercises, may be incorporated. The employment of combined therapeutic modalities may increase the uncontrollability and variability of the studies, making it difficult to understand the effects of acupuncture. We focus solely on pure conventional acupuncture to enhance the stability of the research and better reflect real clinical practice.

Of course, our study also has several limitations. Firstly, the findings are constrained by the fact that only nine Chinese literature sources were ultimately included in the study, this may introduce regional and publication bias. Secondly, many included studies were assessed as having unclear or high risk of bias, particularly regarding randomization, blinding, and allocation concealment. The overall quality of the assessed literature is not high, with a significant risk of publication bias. Thirdly, the short duration of some studies affects the precise therapeutic effect of acupuncture in improving sleep. Fourthly, there is a lack of observation on long-term efficacy, safety and follow-up results in the relevant studies. Fifthly, there is a significant variation in the selection of acupoints and techniques, as well as the frequency, number, and duration of treatments across the studies. The present study has the aforementioned issues, which increase the heterogeneity between similar studies, adversely affect the quality of the research, and also indicate that current research on acupuncture treatment for perimenopausal insomnia is not in-depth. In Chinese populations acupuncture is a widely accepted traditional therapy, so high expectancy may amplify placebo effects and inflate the observed effect size. Confucian-influenced doctor–patient relationships are typically hierarchical, which can enhance adherence and further improve outcomes. Most included trials selected acupoints and stimulation parameters derived from classical Chinese medical theory, and these differ substantially from protocols commonly used in Western settings, potentially leading to divergent results. Given these considerations, we emphasize that caution is required when extrapolating our findings to non-Chinese populations and call for future multicentre trials in culturally diverse settings to confirm the generalizability of acupuncture for perimenopausal insomnia.

## 5 Conclusion

The evidence from this study suggests that the effect of acupuncture therapy in improving perimenopausal insomnia is comparable to that of Western medicine, and acupuncture treatment is significantly effective for patients with perimenopausal insomnia. However, the quality of existing literature is not high, and higher-quality randomized controlled trials are needed to enhance the level of clinical research on acupuncture treatment for perimenopausal insomnia. Furthermore, the execution of large-sample, multi-center, long-term follow-up trials is imperative to obtain more reliable results. Considering the particularities of acupuncture treatment, actively constructing a real-world acupuncture clinical research paradigm will bring more authentic, rich, and practical research outcomes for clinical practitioners.

## Data Availability

The original contributions presented in the study are included in the article/supplementary material, further inquiries can be directed to the corresponding author.
